# Supplementary material for: Evaluation of Sample Preparation Methods for the Analysis of Reef-Building Corals Using 1H-NMR-Based Metabolomics
Source: Metabolites. 2019 Feb 13;9(2):32. doi: 10.3390/metabo9020032 (PMC6410050; doi:10.3390/metabo9020032)
Supplement: Supplementary file 1 [file metabolites-09-00032-s001.pdf]

Supplementary Materials for

Evaluation of sample preparation methods for the analysis of reef-building corals by ^{1}H NMR based metabolomics

Erik Andersson[1,2], Rusty D. Day[2,4], Julie M. Loewenstein[1], Cheryl M. Woodley[3], and Tracey B. Schock[2]

[1] The Graduate School at the University of Charleston, Charleston, SC 29412, USA
[2] National Institute of Standards and Technology, Chemical Science Division, Hollings Marine Laboratory, Charleston, SC 29412, USA
[3]National Oceanic and Atmospheric Administration, National Ocean Service, Hollings Marine Laboratory, Charleston, SC 29412, USA
4Marine Science and Nautical Training Academy (MANTA), 520 Folld Rd Ste 25 PMB 290, Charleston, SC 29412, USA

Figure S1. (a) Representative ^{1}H NMR spectra and (b) PCA scores plot from the three extraction methods for frozen homogenates of both coral species. Metabolic profiles are relatively consistent within species, with slightly higher peak intensities from the methanol extraction (↑). Spectra are normalized to chemical shift standard TMSP at 0.0 ppm. ACER = *Acropora cervicornis*; OFAV = *Orbicella faveolata*; BD = Bligh and Dyer extraction; Methanol = methanol extraction; MTBE = Methyl tert-butyl ether extraction.

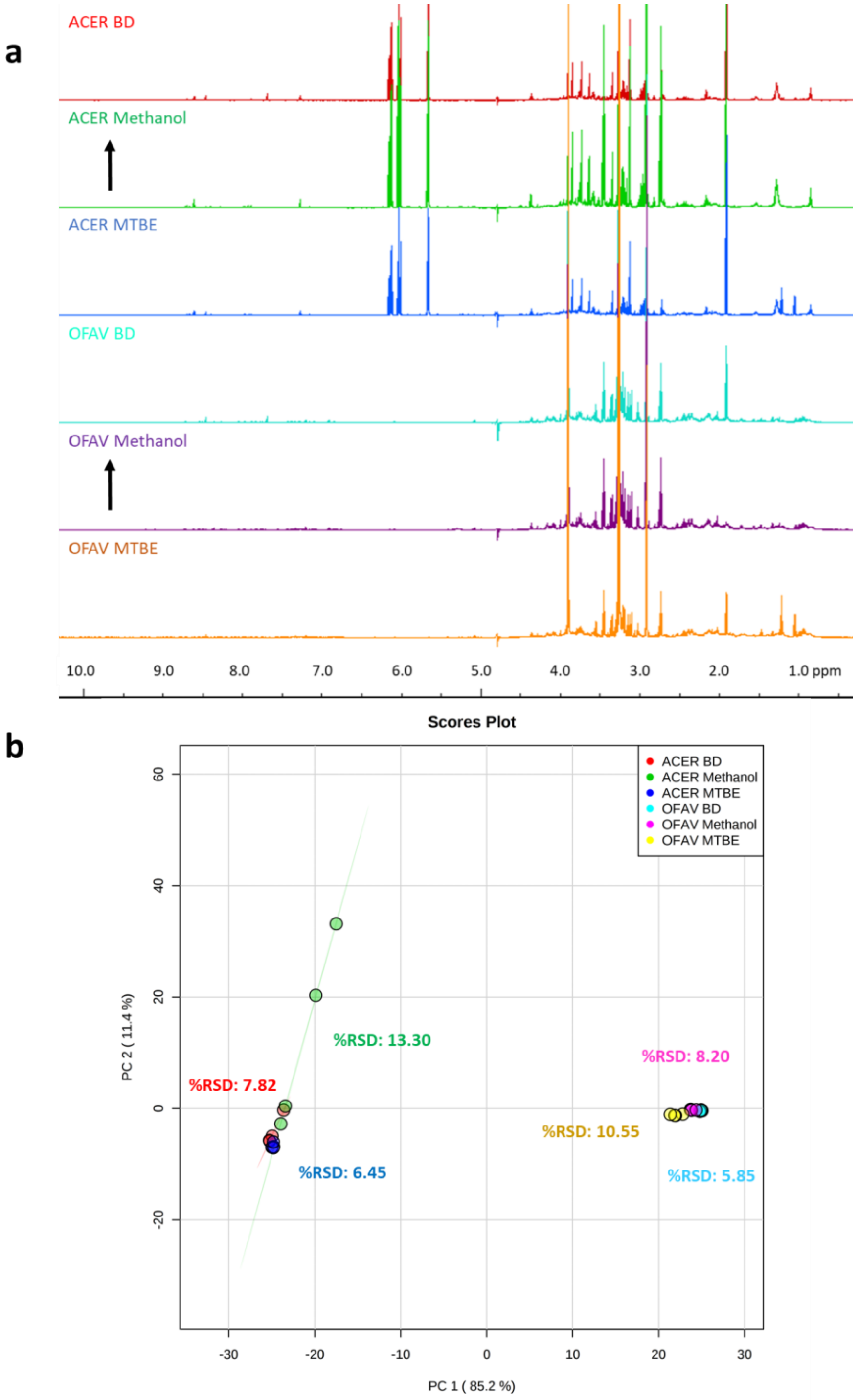

Figure S2. Representative ^{1}H NMR spectra from lyophilized (black) and frozen (blue) *Orbicella faveolata* homogenates demonstrate visually similar metabolic profiles. Metabolites were extracted using the Bligh and Dyer extraction method. Spectra are normalized to chemical shift standard TMSP at 0.0 ppm.

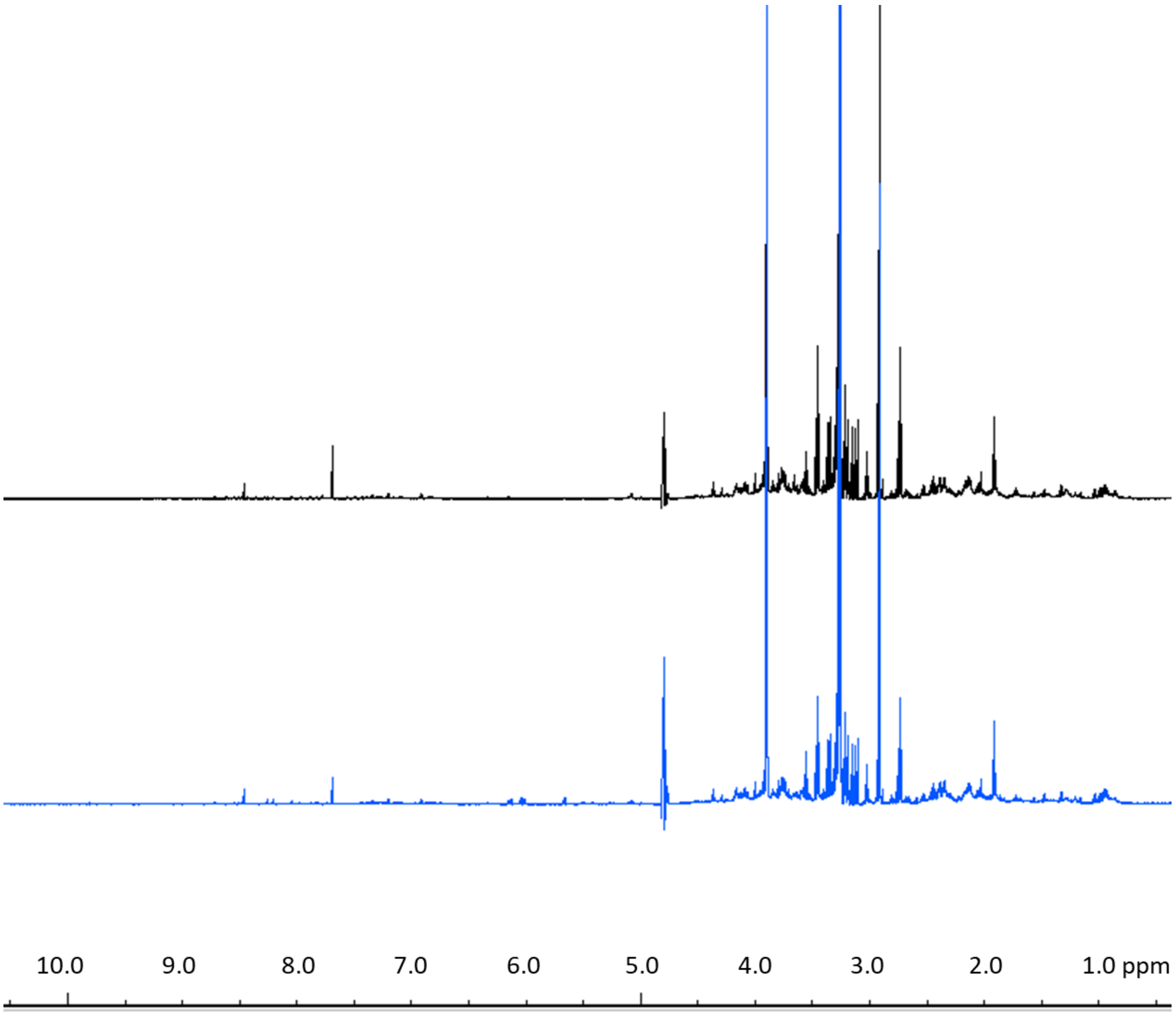

Figure S3. Representative ${}^{1}H$ NMR spectra from nubbin (black) and tissue powder (blue) subsampling methods for unaffected *Porites compressa* samples shows much higher peak intensities resulting from the nubbin method. Samples were lyophilized and metabolites extracted using Bligh and Dyer extraction method. Spectra are normalized to chemical shift standard TMSP at 0.0 ppm.

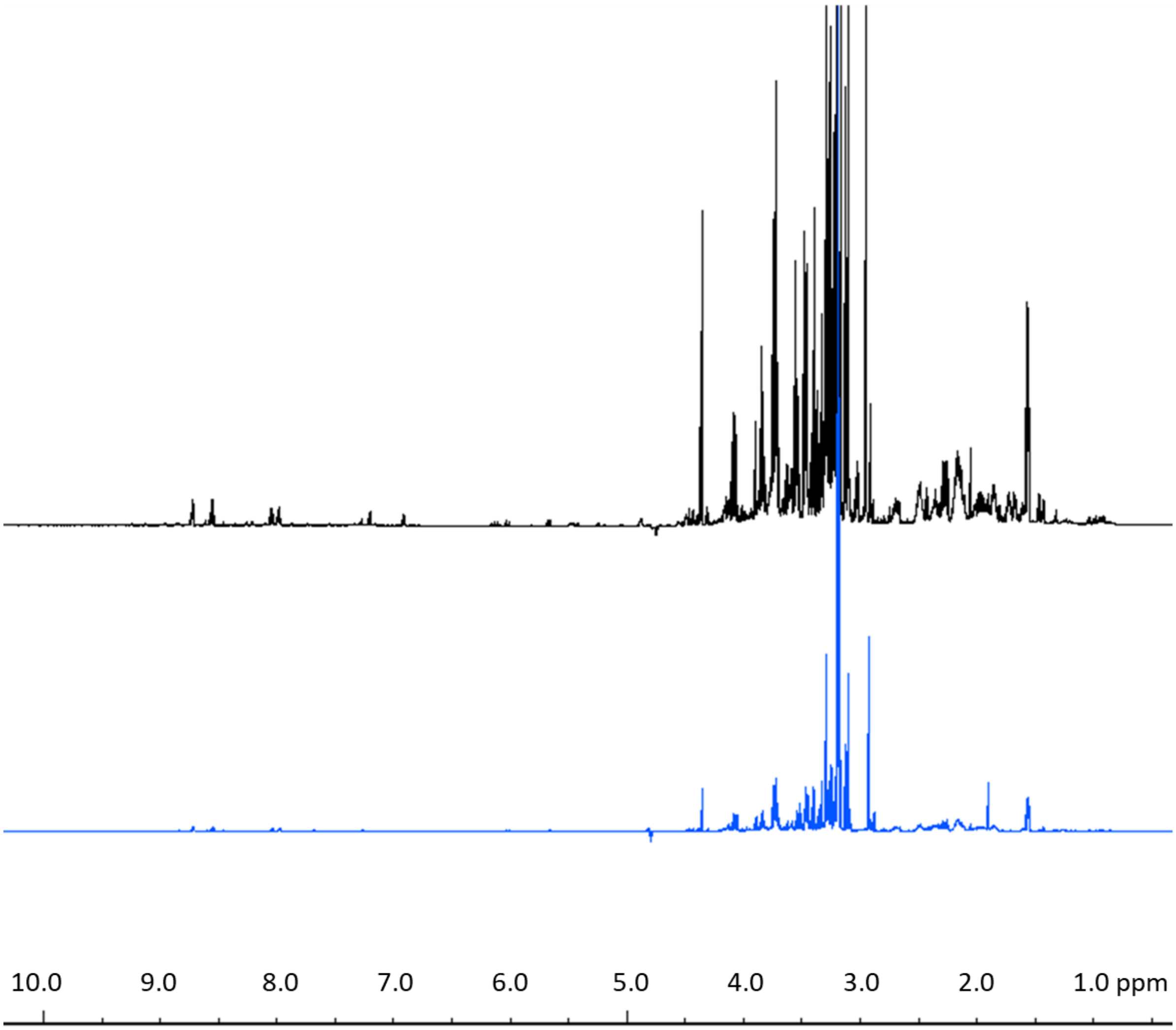

Figure S4. (a) Representative *Porites compressa* ^{1}H NMR spectra of growth anomaly (black) and unaffected (blue) samples and (b) overlayed spectra region from technical replicates of a growth anomaly (black, n = 3) and unaffected (blue, n = 3) *P. compressa* sample. Although overall profiles appear similar, arrows indicate examples of features that differ in intensity between the two samples. Data were collected according to the recommended workflow developed from the current study: Samples were lyophilized, subsampled using the tissue powder method, and metabolites extracted using Bligh and Dyer extraction method. Spectra are normalized to chemical shift standard TMSP at 0.0 ppm.

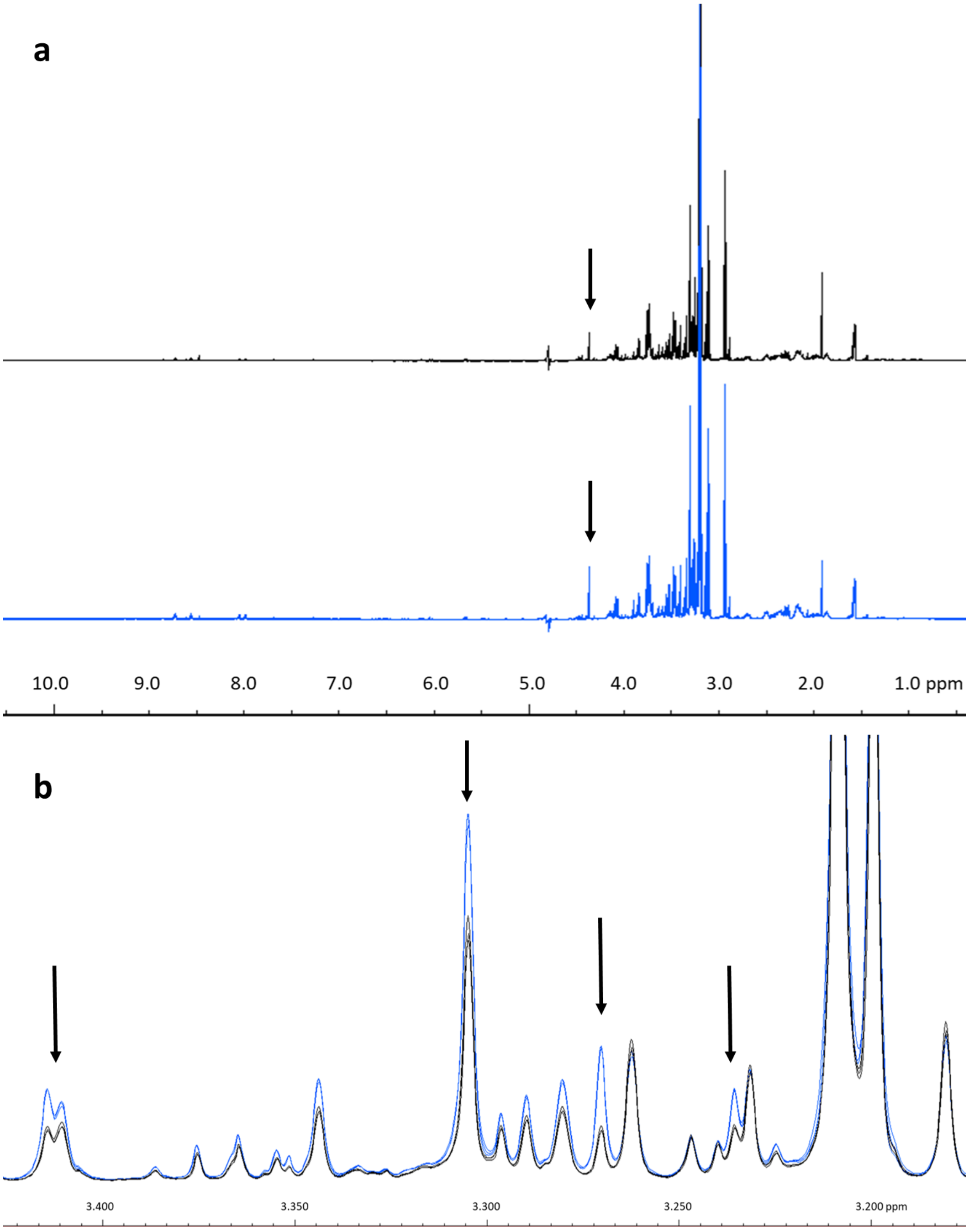

Figure S5. Diagram of processing steps for coral nubbin and tissue powder subsampling methods.

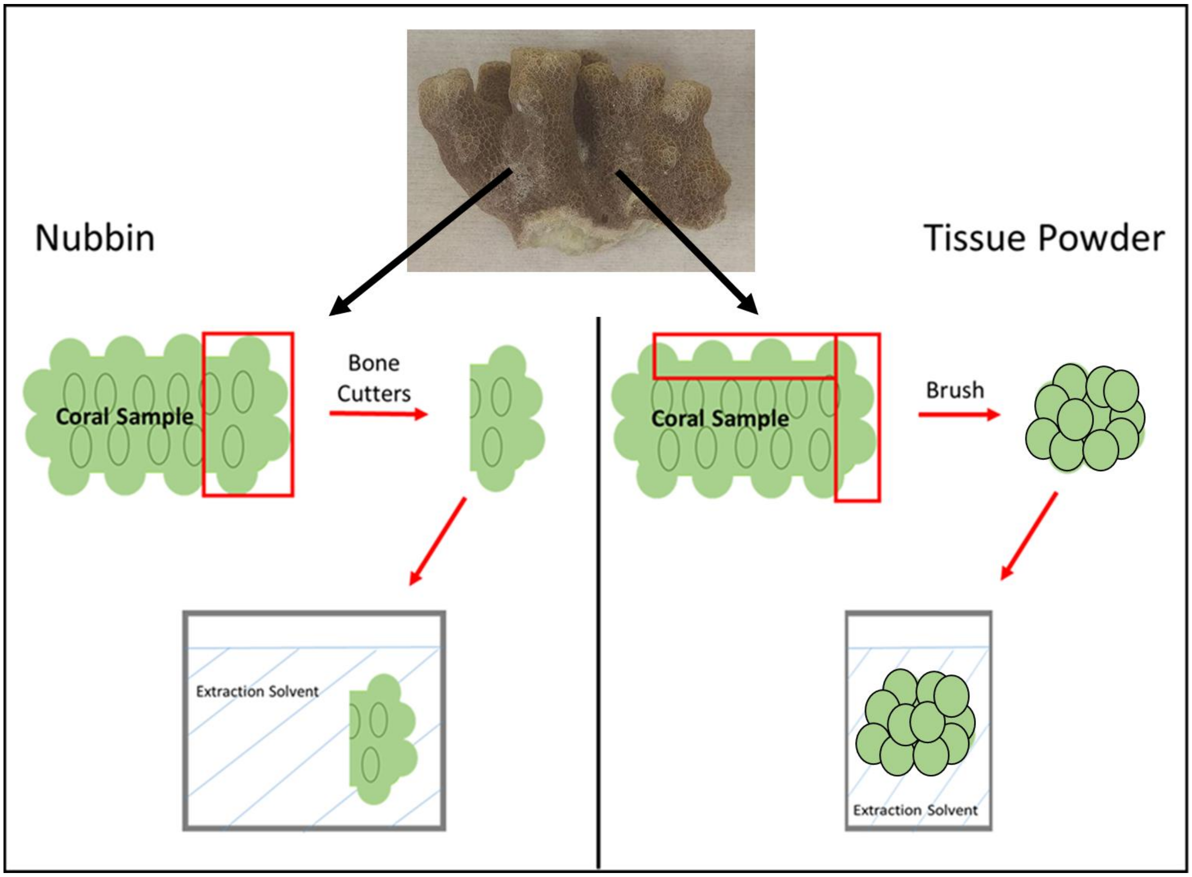

Table S1. Sample preparation methods from published coral metabolomics studies. LN_2 = liquid nitrogen; Chl = chloroform; MeOH = methanol; NMR = nuclear magnetic resonance spectroscopy; LC-MS = liquid chromatography-mass spectrometry, LC=MS/MS = liquid chromatography-tandem mass spectrometry; GC-MS = gas chromatography-mass spectrometry; AASP = *Acropora aspera*; APOC = *Astrangia poculata*; MAEQ = *Montipora aequituberculata*; MCAP = *Montipora capitata*; PACU = *Pocillopora acuta*; PDAM = *Pocillopora damicornis*; PMEA = *Pocillopora meandrina*; PIRR = *Porites irregularis*; PLOB = *Porites lobata*; PRUS = *Porites rus*; SHYS = *Seriatopora hystrix*.

Study	Quenching	Storage	Preservation	Subsampling	Extraction	Analytical Platform	Species Included
Current	LN_2	-80 ℃	Lyophilized	Tissue powder	Chl: MeOH: water	NMR	ACER, OFAV, PCOM,
Gordon *et al.* 2013	LN_2	-80 ℃	Lyophilized	Nubbin	70% MeOH	NMR + LC-MS	AASP,
Parkinson and Baums 2014	LN_2	Not stated	Not stated	Not stated	acetonitrile: isopropanol: water	LC-MS	APOC
Sogin *et al.* 2014	LN_2	-80 ℃	Lyophilized	Nubbin	70% MeOH	NMR	MAEQ, PCOM, PDAM, PLOB, SHYS
Sogin *et al.* 2016	LN_2	Not stated	Not stated	Homogenized	acetonitrile: isopropanol: water	GC-MS	PDAM
Quinn *et al.* 2016	Not stated	Not stated	Not stated	Nubbin	70% MeOH	LC-MS/MS	*Acropora* sp., *Montipora* sp., *Pocillopora* sp., *Porites* sp.
Putnam *et al.* 2016	Lyophilized	-80 ℃	Lyophilized	Nubbin	70% MeOH	NMR	MCAP, PDAM
Hillyer *et al.* 2017	LN_2	-80 ℃	Frozen	Airbrushed	100% MeOH	GC-MS	AASP

Sogin *et al*. 2017	LN_2	-80 ℃	Lyophilized	Nubbin	70% MeOH	NMR	MAEQ, PACU, PIRR, PLOB, PMEA, PRUS, *Acropora* sp., *Montipora* sp.
Hartmann *et al*. 2017	Not stated	Not stated	Not stated	Nubbin	70% MeOH	LC-MS/MS	*Acropora* sp., *Montipora* sp., *Pocillopora* sp., *Porites* sp.
Hillyer *et al*. 2018	LN_2	-80 ℃	Frozen	Airbrushed	100% MeOH	GC-MS	AASP

Table S2. Complete list of solvent volumes used for metabolite extraction from all three experiments: extraction method comparison, metabolism preservation comparison, and subsampling method comparison. MTBE = Methyl tert-butyl ether; *A. cervicornis* = *Acropora cervicornis*; *O. faveolata* = *Orbicella faveolata*; *P. compressa* = *Porites compressa*.

		Polar Solvent System		Non-polar Solvent System			
**Extraction**	**Species**	Methanol (µl)	Water (µl)	Chloroform (µl)	MTBE (µl)	Water (µl)	Total Solvent (µl)
Bligh and Dyer	*A. cervicornis*	321	128	321	-	161	931
	O. faveolata	404	162	404		202	1,172
MTBE	*A. cervicornis*	225	75	-	750	187	1,237
	O. faveolata	225	75	-	750	187	1,237
Methanol	*A. cervicornis*	700	300	-	-	-	1,000
	O. faveolata	700	300	-	-	-	1,000
**Metabolite Preservation**							
Frozen	*P. compressa*	311	125	311	-	156	903
Lyophilized	*P. compressa*	375	150	375	-	188	1,088
**Subsampling**							
Nubbin	*P. compressa*	3,000	1,200	3,000	-	1,500	8,700
Tissue Powder	*P. compressa*	400	160	400	-	200	1,160

Table S3. List of ^{1}H NMR spectral exclusion regions as determined from blank samples. BD = Bligh and Dyer extraction; MTBE = Methyl tert-butyl ether.

	Excluded Range (ppm)	Putative Compound ID	Peak Multiplicity
**Extraction**			
MTBE	1.05-1.07	unknown	doublet
MTBE	1.22-1.23	MTBE	singlet
BD, MTBE	1.91-1.92	acetate	singlet
MTBE	3.23-3.24	MTBE	singlet
BD, MTBE, methanol	4.7-5.0	water	distortion
BD	7.67-7.68	chloroform	singlet
BD, MTBE	8.45-8.46	formic acid	singlet
**Metabolism Preservation**			
Frozen, Lyophilized	1.90-1.93	acetate	singlet
Frozen, Lyophilized	3.23-3.29	unknown	singlet
Frozen, Lyophilized	3.34-3.36	methanol	singlet
Frozen, Lyophilized	4.7-5.0	water	distortion
Frozen, Lyophilized	7.66-7.70	chloroform	singlet
Frozen, Lyophilized	8.45-8.47	formic acid	singlet
**Subsampling**			
Nubbin, Tissue Powder	1.91-1.92	acetate	singlet
Nubbin, Tissue Powder	4.7-5.0	water	distortion
Nubbin, Tissue Powder	8.45-8.46	formic acid	singlet

Table S4. Comparison of *Porites compressa* intra-colony and technical reproducibility in the literature to the current study. Chl = chloroform; MeOH = methanol.

					Spectral %RSD	
**Study**	**Species**	**Preservation**	**Subsampling**	**Extraction**	**Intra**	**Technical**
Current	*P. compressa*	Lyophilized	Tissue powder	Chl: MeOH: water	-	5.7
Current	*P. compressa*	Lyophilized	Nubbin	Chl: MeOH: water	18.4	-
Sogin *et al.* 2014	*P. compressa*	Lyophilized	Homogenized	70% MeOH	-	14.2
Sogin *et al.* 2014	*P. compressa*	Lyophilized	Nubbin	70% MeOH	15.2	-
